# Direct observation of the nanoscale Kirkendall effect during galvanic replacement reactions

**DOI:** 10.1038/s41467-017-01175-2

**Published:** 2017-10-31

**Authors:** See Wee Chee, Shu Fen Tan, Zhaslan Baraissov, Michel Bosman, Utkur Mirsaidov

**Affiliations:** 1 Department of Physics, National University of Singapore, Singapore, 117551 Singapore; 2 Centre for Bioimaging Sciences and Department of Biological Sciences, National University of Singapore, Singapore, 117557 Singapore; 3 Centre for Advanced 2D Materials and Graphene Research Centre, National University of Singapore, Singapore, 117546 Singapore; 4 Institute of Materials Research and Engineering, A*STAR (Agency for Science, Technology and Research), Singapore, 138634 Singapore; 5 Department of Materials Science and Engineering, National University of Singapore, Singapore, 117575 Singapore; 6 NUSNNI-NanoCore, National University of Singapore, Singapore, 117411 Singapore

## Abstract

Galvanic replacement (GR) is a simple and widely used approach to synthesize hollow nanostructures for applications in catalysis, plasmonics, and biomedical research. The reaction is driven by the difference in electrochemical potential between two metals in a solution. However, transient stages of this reaction are not fully understood. Here, we show using liquid cell transmission electron microscopy that silver (Ag) nanocubes become hollow via the nucleation, growth, and coalescence of voids inside the nanocubes, as they undergo GR with gold (Au) ions at different temperatures. These direct in situ observations indicate that void formation due to the nanoscale Kirkendall effect occurs in conjunction with GR. Although this mechanism has been suggested before, it has not been verified experimentally until now. These experiments can inform future strategies for deriving such nanostructures by providing insights into the structural transformations as a function of Au ion concentration, oxidation state of Au, and temperature.

## Abstract (web summary)

Hollow nanoparticles can be synthesized by galvanic replacement or the Kirkendall effect, which are generally regarded as two separate processes. Here, the authors use liquid TEM to follow the entire galvanic replacement of Ag nanocubes, finding experimental evidence that the Kirkendall effect is a key intermediate stage during hollowing.

## Introduction

Hollow nanomaterials are important candidate materials for many technological applications^1^, such as catalysis^2–5^, energy conversion^6, 7^, and medicine^8, 9^, because of their high surface-to-volume ratio, effective mass transport of reactants to their solid surfaces and enhanced plasmonic properties^10^, when compared to their solid counterparts. Galvanic replacement^11^ (GR) reactions are commonly used to synthesize such structures. The underlying chemistry is straightforward; an electrochemical reaction takes place when a metal ion having a higher reduction potential (more noble) is in contact with another metal having a lower reduction potential within an electrolyte, which leads to the preferential corrosion of the second metal. Hence, when a noble metal salt solution is introduced to nanoparticles made of a metal with lower reduction potential, the noble metal deposits around a nanoparticle as the nanoparticle template is concurrently removed from the inside out through gaps in the deposited layer^11^. If the noble metal layer encapsulates the template, hollow nanostructures are formed^12^. Morphology and composition of these structures can be controlled by tuning the synthesis conditions^13^, such as shape of the metal template^14–16^, temperature^17^, and concentrations of the relevant ions^16, 17^.

Our current understanding of how these hollow nanostructures evolve is largely derived from “quench-and-look” studies, where the reaction mixture is quenched after adding incremental amounts of reactants, followed by an examination of the reaction products^16–19^. However, short-lived intermediate states are difficult to capture with this approach. Recent studies combining in situ single particle optical spectroscopy and ex situ electron microscopy^20–22^ suggest that the mechanism behind these hollow structures may be more complex than that expected from straightforward electrochemical dissolution. For example, Smith et al.^20^ observed that Ag nanoparticles exposed to Au^3+^ ions displayed non-linear reaction kinetics during GR. They monitored the scattering intensity of individual Ag nanoparticles and found that it always decreased abruptly over a few seconds. This behavior was explained with a model where the nucleation of Ag vacancies into a critical void was a pre-requisite for hollowing. It is also possible to image the reaction directly with in situ microscopy and capture the complete evolution of individual nanoparticles during a reaction for a better understanding of these transient processes, but previous work with electron^23–25^ or X-ray^26^ imaging had certain experimental limitations. For one, typical reaction protocols require elevated temperatures^27^, but the microfluidic systems used in those earlier studies did not allow heating of the reaction cell. The slow rate of reaction at room temperature can also cause the experiment to be susceptible to artifacts due to the radiolysis of reactants by the energetic beams used for imaging^23^. Furthermore, the imaging rates used may not be sufficient to capture fast reaction dynamics (one frame every 2 s in ref. ^26^. and one frame every 4.2 s in refs. ^23, 25^).

There is a need for better understanding of the mechanism(s) behind the morphological evolution. It has been proposed that GR and the Kirkendall effect (KE) can couple and lead to more complex hollow structures when the rate of GR is reduced by performing the synthesis at room temperature and with a modified solution chemistry^28^. KE arises from unequal diffusion rates between two interdiffusing atomic species generating a net vacancy flux, which leads to void formation near the two-material interface^29^. Metal nanoparticles can turn into hollow nanostructures during oxidation if the outward flux of atoms from the core is larger than the inward flux of atoms from the shell^30–32^. Recent studies suggest that the effect of KE may manifest itself in GR reactions at higher temperatures^33^ and in bimetallic nanostructures^34^.

Here, we use liquid cell transmission electron microscopy^35, 36^ (TEM) to reveal how individual poly(vinyl)pyrrolidone (PVP)-coated Ag nanocubes (~75 nm in size) evolve into hollow structures during GR with Au ions. In our experiments, we access different reaction regimes by tuning the reaction temperature from 23 to 90 °C and by changing the oxidation state of Au. The replacement reactions are initiated by flowing an aqueous solution containing Au ions into the liquid cell through the external fluid channels. Electron detection using a fast and sensitive CMOS camera allows us to lower the incident electron flux to <30 e^−^ per (Å^2^ s), while maintaining an acquisition rate of 25 frames per second. These low-dose conditions minimize perturbation of the observed reactions by the electron beam^37^ (Supplementary Note 2). Our observations indicate that the transition to a hollow structure is due to the nucleation and propagation of voids within the nanocubes, and imply the existence of an intermediate transformation based on KE. These results suggest that KE indeed couples to GR in the reaction between Ag and Au, and that the coupling is observed generally across different reaction chemistries and conditions.

## Results

### Galvanic replacement reactions with Au^3+^

The overall reaction for the first precursor, chloroauric acid (HAuCl_4_), reacting with Ag is as follows:

1 $$3{\rm{Ag}}\left( {\rm{s}} \right) + {\rm{AuCl}}_4^ - \left( {{\rm{aq}}} \right) \to {\rm{Au}}\left( {\rm{s}} \right) + 3{\rm{AgCl}}\left( {\rm{s}} \right) + {\rm{C}}{{\rm{l}}^ - }({\rm{aq}})$$

The standard reduction potential of ${\rm{AuCl}}_4^ - {\rm{/Au}}$ (0.99 V vs. standard hydrogen electrode, SHE) is higher than that of ${\rm{AgCl} }{\rm{/Ag}}$ (0.22 V vs. SHE), which results in the spontaneous replacement of three Ag atoms by a deposited Au atom.

**Fig. 1:**
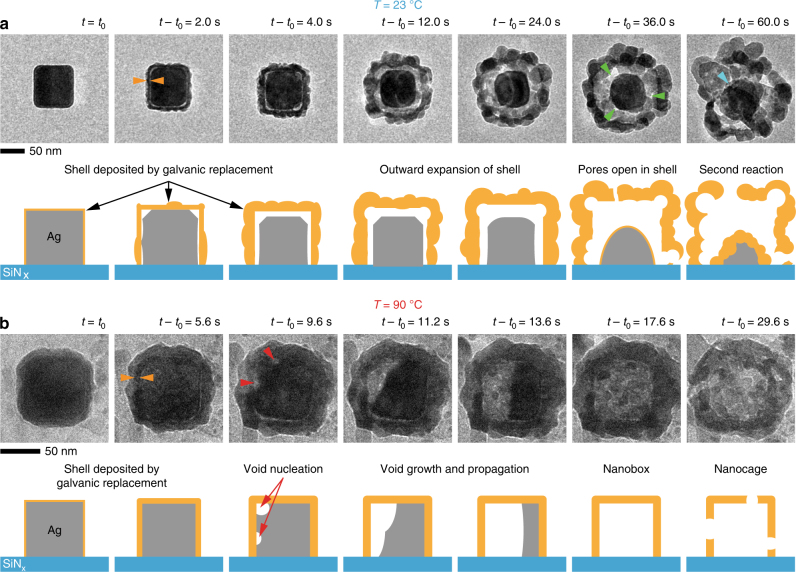
Galvanic replacement of Ag nanocubes by Au at 23 and 90 °C. **a** Time series of in situ transmission electron microscopy (TEM) images (Supplementary Movie 1) and corresponding schematics showing the morphological evolution of an Ag nanocube during galvanic replacement (GR) reaction at 23 °C. Green arrows indicate pores that form in the deposited shell, and a cyan arrow points to a second GR reaction on the residual Ag core (formation of a rough shell) after it is again exposed to the Au solution through pores on the outer shell. **b** Time series of in situ TEM images (Supplementary Movie 2) and schematics showing the morphological evolution of an Ag nanocube during GR at 90 °C. Orange arrows indicate the inner shell with darker contrast, which should be the Au layer. Red arrows indicate observed void nucleation. Here, *t*_0_ indicates the start of the recording time

**Fig. 2:**
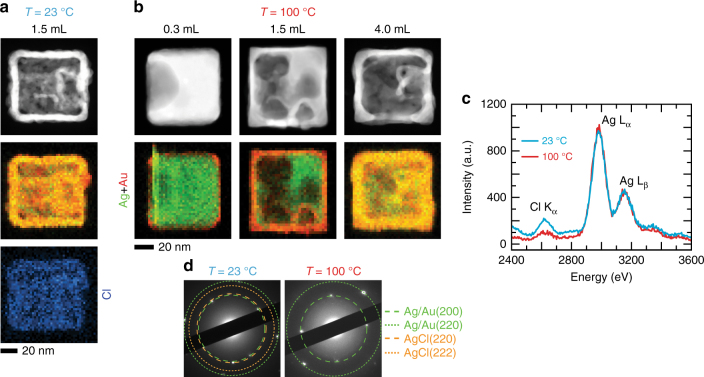
Chemical and structural characterization of Ag/Au nanostructures synthesized ex situ. **a** Scanning TEM annular dark field image and corresponding energy dispersive X-ray spectroscopy (EDX) chemical maps of nanostructures synthesized ex situ at 23 °C. **b** Nanostructures synthesized at 100 °C after adding 0.3, 1.5, and 4.0 mL of 0.1 mM HAuCl_4_. **c** EDX spectra for samples synthesized at 23 and 100 °C (with 1.5 mL of 0.1 mM HAuCl_4_ added) showing the Cl K and Ag L lines. **d** Selected area electron diffraction from a single nanostructure in both samples (with 1.5 mL of 0.1 mM HAuCl_4_ added), where diffraction spots for AgCl^44^ are frequently found in the 23 °C sample because of lower solubility of AgCl at 23 °C than at 100 °C

In Fig. 1, we compare the image sequences describing the morphological evolution recorded in situ at 23 °C (Supplementary Movie 1) and 90 °C (Supplementary Movie 2). At 23 °C, the Ag nanocubes are quickly encapsulated by a thin layer of material (Fig. 1a: *t* − *t*_0_ = 2.0 s), which should be a mixture of Au and precipitated AgCl^17^ (Fig. 2). As the reaction progresses (Fig. 1a: *t* − *t*_0_ = 4.0 s), the outer layer gets thicker and voids form at the corners of the Ag nanocubes. The rough surface is caused by the precipitation of AgCl interfering with epitaxial growth of Au^17^. By *t *− *t*_0_ = 12.0 s (Fig. 1a), a clear gap can be seen between the nanocube and the deposited layer. The deposited layer expands outwards and becomes more corrugated with time (Fig. 1a: *t *− *t*_0_ = 36.0 s), while a significant portion of the original Ag nanocube remains within. This result is surprising because the replacement reaction should continue until all the Ag is depleted. It is worth mentioning here that the need for an interface of two materials and nucleation of multiple voids at said interface are more commonly recognized as hallmarks of hollowing via KE^31, 32^. Beyond *t* − *t*_0_ = 36.0 s, pores appear as the outer shell collapses, and we observe a second GR reaction taking place on the residual core (Fig. 1a: *t* − *t*_0_ = 60.0 s). We will discuss the significance of these observations further after we look at the results obtained at 90 °C.

At 90 °C (Fig. 1b), we can discern two levels of contrast in the shell surrounding the nanocube after *t* − *t*_0_ = 5.6 s; a darker inner shell that should be deposited Au based on mass thickness contrast (highlighted with orange arrows in Fig. 1b, also see Fig. 2) and surrounding it, a lighter outer shell. This outer layer is likely AgCl that has formed during the initial oxidation of Ag^22,19^ by Au because the relatively thin liquid layers in the liquid cell cannot effectively solubilize the AgCl formed during the reaction (see Supplementary Note 3). At *t* − *t*_0_ = 9.6 s (Fig. 1b), two voids form in the upper left corner of the nanocube and the interface between the inner shell and the Ag nanocube (red arrows), which then merge into a single larger void (Fig. 1b: *t* − *t*_0_ = 11.2 s). The void propagates across the nanocube (Fig. 1b: *t* − *t*_0_ = 13.6 s) until the structure is completely hollow (Fig. 1b: *t* − *t*_0_ = 17.6 s). Note that the nanocube maintains contact with the two-metal interface until it disappears completely. Eventually, large pores appear in the walls, which lead to degradation of the entire structure (Fig. 1b, *t* − *t*_0_ = 29.6 s).

Here, we highlight three important features of these in situ observations. First, the Ag nanocube cores appear to be largely intact until the voids nucleate (Fig. 1a: *t* − *t*_0_ = 2.0 s and Fig. 1b: *t* − *t*_0_ = 9.6 s). Then, Ag is removed through the growth of these voids. We propose that the voids are Kirkendall voids and that the vacancies forming these voids are created as GR continues to take place on the surface of the nanostructures, adding more Au to the surface while depleting it of Ag at the same time. This process creates a sustained concentration gradient that causes Ag atoms to diffuse into and through the encapsulating layer into the solution. The formation and growth of voids on the Ag side are also consistent with the diffusion of Ag into Au being faster than the diffusion of Au into Ag and with earlier reports of Kirkendall voids in Ag for bulk Ag–Au diffusion couples^38, 39^. Second, the voids always nucleate at the interface between Ag and Au and near the corners. The preference for void nucleation at the cube corners can be explained by them being high surface energy regions^20^, whereas the propensity to nucleate fewer voids at elevated temperature is due to higher vacancy diffusion rates, which allow vacancies to travel and add to existing voids. Moreover, since the addition of vacancies to an existing void is energetically favorable compared to the nucleation of new voids, the two-metal interface can be maintained until the nanostructure is completely hollow on the inside. In contrast, at 23 °C, multiple voids nucleate at the interface, creating the gap that separates the two metals, and the removal of Ag slows down. More importantly, we observe a second galvanic replacement reaction only when pores open up in the shell, indicating that the shell is impermeable to the Au solution. Third, we find that AgCl always precipitates around the Ag nanocubes as the Ag core becomes hollow (Supplementary Figs. 8 and 9). These by-products of Ag dissolution do not correlate with the location of voids, which implies that Ag atoms leave the core in an isotropic manner. It should be noted that these results differ from the observations of nanoparticle dissolution during etching, where the nanoparticles continuously shrink until they disappear^40, 41^.

Next, we compare these observations with the commonly accepted sequence of morphological evolution for GR of Ag nanocubes^11^, which is summarized as follows. At the beginning, Au gradually covers the Ag nanocube, leaving a single pinhole on one of the {100} sidewalls through which Ag is removed by Au and a corresponding cavity forms beneath the pinhole^33^. Further reaction leads to the formation of a structure that is hollow on the inside (a so-called nanobox^14^), where the continuous Au layer is transformed into an Au–Ag alloy due to interdiffusion between Au and Ag. Subsequently, dealloying of Au–Ag results in pore formation in the alloy layer (a nanocage^18^) and finally, breakdown of the nanostructure into smaller particles. It is commonly assumed that material removal during GR occurs primarily through the pinholes in the shells^42^, but our in situ experiments indicate that pinholes are not the only pathway by which Ag is removed.

In our previous study on the GR of Ag nanocubes by Au ions^24^, we showed that Au deposits on the corners of the nanocube at the early stages and that the nanocube sidewalls are etched into bowl-shaped cavities. However, we did not observe the interior become hollow in those experiments because the static liquid cell we used could only contain a very small volume of Au precursor solution. There, we also had to modify the Au solutions with ethylenediaminetetraacetic acid to slow down the reaction, or else, the nanocubes would have reacted within the 5 min needed to transfer the sample into the TEM^24^. In the current experiments, the flow cell allows us to initiate the reaction controllably. Liquid flow also replenishes the Au precursor solution and offsets the depletion of Au ions by solvated electrons^41^, which are created when water molecules interact with high energy electrons and undergo radiolysis^43^. The lower electron flux of <30 e^−^ per (Å^2^ s) compared to our earlier study (2000–5000 e^−^ per (Å^2^ s))^24^, further reduces such radiolytic effects (Supplementary Note 2). It means that we now raise the amount of available Au ions to much higher levels when compared to the earlier experiments. Therefore, the reaction proceeds beyond the initial stages, and we see a complete protective shell forming on the nanocubes within a few seconds. On the account of both studies, we conclude that the removal of Ag from the core occurs by both electrochemical dissolution and Kirkendall void formation. In addition, our results do not support the conventional picture of Ag removal via a single dominant pinhole, although we do not rule out the possibility of Ag transport through multiple nanoscopic pores in the encapsulating shell.

### Characterization of structures synthesized ex situ

We reiterate here that the conventional description for GR is derived from ex situ studies, which are not sensitive to transient reaction stages. It is also challenging to make definitive conclusions about the mechanism of void formation from these studies because the comparisons are made using randomly picked particles from the different reaction conditions. Figure 2 shows nanostructures obtained using “quench-and-look” after different amounts of HAuCl_4_ had been added at 23 and 100 °C, and following standard in-flask protocols for GR^27^. A saturated NaCl solution wash was used to remove AgCl from the extracted solutions^27^. Then, these samples were characterized with scanning electron microscopy (SEM), scanning TEM, energy dispersive X-ray (EDX) spectroscopy, and electron diffraction (Supplementary Note 1). Figure 2a, b depicts the scanning TEM images and EDX maps of nanostructures synthesized at 23 and 100 °C, respectively. EDX analysis (Fig. 2c) indicates that a higher concentration of Cl is inherent to the 23 °C sample when compared with the 100 °C sample. Selected area diffraction patterns (Fig. 2d) also show diffraction spots for AgCl^44^ in the 23 °C samples, whereas the 100 °C samples only show spots for Ag/Au (Ag and Au have similar reciprocal spacing between lattice planes). These results are consistent with previous work that reports co-deposition of AgCl and Au during GR at room temperature because of the decreased solubility of AgCl at lower temperatures^17^.

Figure 2b also shows the nanostructures obtained at 100 °C after adding different amounts of HAuCl_4_. In the first pair of images (Fig. 2b: 0.3 mL of 0.1 mM HAuCl_4_), the scanning TEM image shows a cavity on one side of the nanocube, whereas the EDX map confirms Au deposition on the nanocube surface. An extensive void is seen in the Ag nanocube where 1.5 mL of 0.1 mM HAuCl_4_ (Fig. 2b) was added, in conjunction with a thicker layer of Au. According to this chemical map, there is Ag mixed into the Au layer, implying alloying in this outer layer. After 4.0 mL of 0.1 mM HAuCl_4_ (Fig. 2b) was added, we find a box-shaped shell that is entirely hollow on the inside, which consists of both Ag and Au. The atomic percentages of Ag and Au for the three data sets as extracted from EDX maps (Fig. 2b) are provided in Table 1. Additional EDX results are provided in Supplementary Fig. 6 and 7.

**Table 1:** Atomic compositions of nanostructures obtained from galvanic replacement (GR) of Ag nanocubes by Au at 90 °C

**Amount of 0.1 mM HAuCl**_**4**_ **added (mL)**	**Final concentration of Au** ^**3+**^ **ions in solution (µM)**	**Atomic % of Ag in nanostructure (measured with EDX)**	**Atomic % of Au in nanostructure (measured with EDX)**
0.3	5.6	97	3
1.5	23	78	22
4.0	44	67	33

### Comparing in situ and ex situ results

**Fig. 3:**
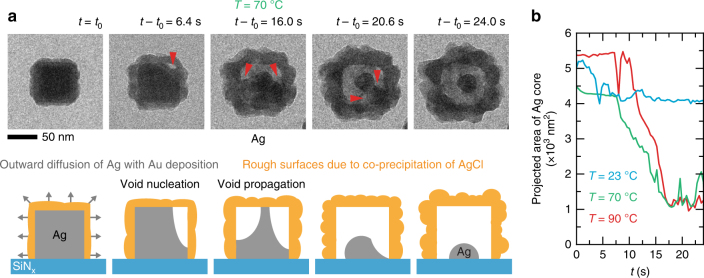
Galvanic replacement of an Ag nanocube by Au at 70 °C. **a** Time series of in situ TEM images (Supplementary Movie 3) and corresponding schematic depicting how the morphological evolution can be explained via the propagation of two or more voids. **b** The projected area of the Ag nanocube cores vs. time at three different temperatures

Next, we show the in situ results obtained at 70 °C in Fig. 3, which exemplify hollowing via Kirkendall void formation. The manner in which the nanostructure becomes hollow can be explained by the intersection of two (or more) voids that start from the corners of the nanocube (Fig. 3a: *t* − *t*_0_ = 16.0 s). However, the outward diffusion of Ag at 70 °C is not fast enough to create a completely hollow structure (compared to 90 °C). Hence, a small portion of Ag remains in the center of the nanostructure (Fig. 3a: *t* − *t*_0_ = 24.0 s). It clearly shows that the structure can only become fully hollow while pathways for Ag diffusion exist (i.e., the core is still in contact with the shell). At *t* − *t*_0_ = 36.0 s, the shell dislodges, and we again observe the remaining Ag core undergo GR (Supplementary Fig. 12). In Fig. 3b, we compare the dissolution rate of the nanocube (in terms of projected area) at the three temperatures over a span of 24 s. Qualitatively, the dissolution rate increases with temperature. The time scales of hollowing, which span over several seconds, are also consistent with the transformation times reported by Smith et al.^20^. These rapid dynamics can be explained by Ag atoms moving along fast diffusion pathways, such as grain boundaries and defects in the outer shell^45^.

Here, we address the cosmetic differences between the in situ and ex situ results. First, as mentioned earlier, the thin liquid conditions in liquid cells promote the precipitation of solid AgCl. In fact, it will be strange if we do not observe AgCl formation, since its presence is expected from the reaction chemistry (Eq. 1) and the low solubility of AgCl in water. Second, the Au ions are introduced continuously in the in situ experiments (i.e., the Au ion concentration in the liquid cell increases), leading to the extended evolution of the nanostructures, whereas in the ex situ synthesis, the reaction is limited by the amount of Au solution added. Nevertheless, we can still associate the room temperature ex situ sample (Fig. 2a) with the in situ intermediates at 23 °C (Fig. 1a: *t* − *t*_0_ = 4.0 s) and 90 °C (Fig. 1b: *t* − *t*_0_ = 9.6 to *t* − *t*_0_ = 13.6 s). Third, in the in situ experiments, the reaction takes place mainly on exposed faces of the nanocube due to contact with a supporting SiN_x_ membrane. Nanostructures observed at 23 °C (Fig. 1a) and 70 °C (Fig. 3) resemble the hut-shaped structures found in substrate-based GR reactions^46, 47^. The remnant Ag cores observed in the in situ experiments do not show up in the ex situ synthesis because the eroding Ag core can move within the hollow structure during conventional colloidal synthesis and stay in touch with the encapsulating shell.

The composition of the reacting solution (capping agents and solvent) may also influence the nanostructure morphology obtained from GR^15, 32^. In Supplementary Fig. 10, we show results where we used ethylene glycol to modify the reaction^48^. The nanocubes are dispersed in a solution containing 1% ethylene glycol (by volume). During GR, we observe two stages of nanocube dissolution, the formation of an initial cavity (which may be due to pinholes), followed by hollowing of the entire nanostructure via the isotropic outward diffusion of Ag. We also discuss results from experiments where we used an Ag etchant^49^, iron nitrate (Fe(NO_3_)_3_) to react with the Au–Ag nanoboxes to form Au nanoframes in Supplementary Note 4.

### Galvanic replacement reactions with Au^+^

**Fig. 4:**
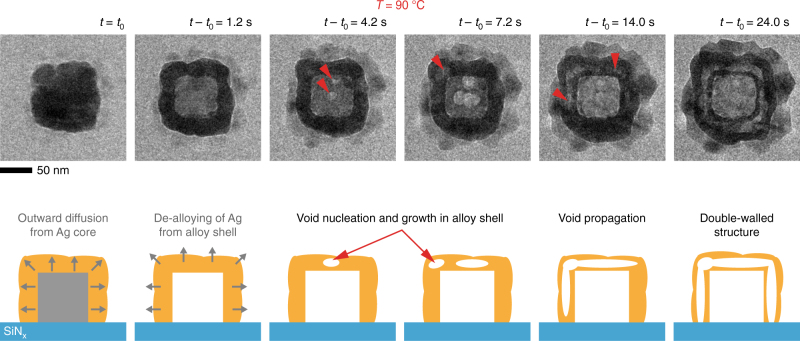
GR of an Ag nanocube using an AuCl precursor solution at 90 °C. Time series of in situ TEM images (Supplementary Movie 4) and the corresponding schematic depicting the creation of double-walled structures via void nucleation and propagation within the alloy shell. Red arrows indicate the nucleation of voids and their propagation within the shell

We further explore the possibility of tuning the removal rate of Ag by using an Au(I) salt as the precursor, instead of an Au(III) salt, while keeping the reaction temperature at ~90 °C. The reaction proceeds as follows:

2 $${\rm{Ag}}\left( {\rm{s}} \right) + {\rm{AuCl}}_2^ - \left( {{\rm{aq}}} \right) \to {\rm{Au}}\left( {\rm{s}} \right) + {\rm{AgCl}}\left( {\rm{s}} \right) + {\rm{C}}{{\rm{l}}^ - }\left( {{\rm{aq}}} \right),$$

where the standard reduction potential for ${\rm{AuCl}}_2^ - {\rm{/Au}}$ of 1.11 V vs. SHE is still higher than that for ${\rm{AgCl} }{\rm{/Ag}}$, with one Ag atom being removed for every Au atom^19^. Here, we observed that the nanocubes form complex nanostructures (Supplementary Movie 4) similar to that observed by González et al.^28^, who employed a more complicated room temperature reaction chemistry. The nanocube shown in Fig. 4 turns into a structure with two parallel walls. After the initial hollowing (Fig. 4: *t* − *t*_0_ = 1.2 s), the two walls are created through the nucleation of voids within the thick outer shell (Fig. 4: *t* − *t*_0_ = 4.2 s, *t* − *t*_0_ = 7.2 s) and their subsequent propagation (Fig. 4: *t* − *t*_0_ = 14.0 s). However, this reaction pathway seems to be rare in chemical synthesis at elevated temperatures. We highlight that such structures were not reported in a study involving colloidal synthesis under similar conditions^19^. Our ex situ synthesis results using Au (I) ions also show few nanostructures with partially and fully formed double parallel walls (Supplementary Fig. 13). We speculate that the scarcity of such structures is due to the higher solubility of AgCl at elevated temperatures (which is offset by the thin liquid conditions found in liquid cells), but the exact mechanism still needs to be determined. Although the in situ movie depicts small differences in the sequence of structural evolution from that suggested by González et al.^28^ from “quench-and-look” (we do not see indications of a dominant pinhole); the idea that KE is an intermediate mechanism by which such hollow structures are created during GR remains consistent. More importantly, these results lend further support to our hypothesis that the coupling between KE and GR can occur over a range of synthesis conditions.

In conclusion, our direct, real-time observations reveal that Ag nanocubes become hollow during galvanic replacement via the nucleation and growth of voids at the interface between Ag and Au, which often starts near the corners of the nanocubes. Transient stages of the structural evolution confirm that these hollow structures form via the coupled action of GR and KE. More importantly, the results indicate that this coupling occurs across different reaction conditions. This study provides clear insights into the nanostructure evolution as a function of Au ion concentration, the oxidation state of Au and temperature, and with those insights, a new mechanistic understanding of the formation of hollow structures during GR. Furthermore, the experiments with ethylene glycol suggest that our approach can be extended to target-specific co-reactants in the synthesis protocols and elucidate their roles in morphology control. Such investigations can help us further tailor synthesis protocols to obtain nanostructures with properties that are optimized for their applications.

## Methods

### Sample preparation

An aliquot of 50 µL of poly(vinyl)pyrrolidone (PVP) stabilized Ag nanocubes (Cat. No. SKU:SCPH75-5M, nanoComposix Inc., San Diego, CA, USA) at a stock concentration of ~2 × 10^11^ nanocubes per mL were first loaded into a 1.5 mL-centrifuge tube. Then, the solution was centrifuged at 10,000 rpm for 5 min and redispersed in deionized water to reduce the concentration of PVP in solution. The gold solutions were prepared from gold(III) chloride trihydrate, HAuCl_4_ (Cat. No. 520918-5G, Sigma-Aldrich Co., St Louis, MO, USA) and gold(I) chloride, AuCl (Cat. No. 481130-1G, Sigma-Aldrich Co., St Louis, MO, USA).

### In situ experiments

The liquid cell is made up of two chips: (1) a heater chip with a 50 nm thick silicon nitride (SiN_x_) membrane window (Hummingbird Scientific, Lacey, WA, USA) and (2) bottom chip with a 25 nm SiN_x_ window (Supplementary Fig. 1) that we fabricated in-house^50^. These chips were first plasma treated before experiments to make their SiN_x_ membrane surfaces hydrophilic, so that liquid can easily wet the interior of the liquid cell. Next, ~500 nL of the nanocube solution was drop casted onto the heater chip and allowed to dry. Then, the liquid cell is assembled within a liquid cell TEM holder (Hummingbird Scientific, Lacey, WA, USA). These procedures are described in detail in the Supplementary Methods.

The holder was loaded into a JEOL 2010FEG TEM (JEOL Ltd., Tokyo, Japan) operated at 200 kV. After heating the Ag nanocubes to the targeted temperature (±10 °C), 1.0 mM of the Au precursor solution was flowed into the cell through the fluid tubing using a syringe pump at a rate of 20 µL per min. Images were recorded with Gatan OneView camera (Gatan Inc, Pleasanton, CA, USA) at a rate of 25 frames per second. The image processing algorithm used to extract the etch rates of nanocubes is described in the Supplementary Methods.

### Ex situ characterization

Samples for ex situ characterization were prepared according to the protocols outlined in ref. ^27^. EDX analysis was performed with an FEI Titan TEM (FEI Company, Hillsboro, OR, USA) with a Schottky electron source operated at 200 kV in scanning TEM (STEM) mode and an EDAX Tops System (EDAX Inc., Mahwah, NJ, USA) with a detector size of 30 mm^2^. An electron probe with an approximate diameter of 0.3 nm was used, and the images were collected using the high-angle annular dark field detector. A 0.5-nm electron probe and an acquisition time of 150 ms were used to collect each spectrum. The SEM images were collected with an FEI Verios 460 field emission SEM operated at 2 kV. Further details for the ex situ experiments can be found in the Supplementary Methods.

### Data availability

The data reported in this study are available from the corresponding author on reasonable request.

## Electronic supplementary material

Supplementary Information

Description of Additional Supplementary Information

Supplementary Movie 1

Supplementary Movie 2

Supplementary Movie 3

Supplementary Movie 4
