# Supplementary material for: Direct observation of the nanoscale Kirkendall effect during galvanic replacement reactions
Source: Nat Commun. 2017 Oct 31;8:1224. doi: 10.1038/s41467-017-01175-2 (PMC5663914; doi:10.1038/s41467-017-01175-2)
Supplement: Supplementary file 1 — Supplementary Information [file 41467_2017_1175_MOESM1_ESM.pdf]

Supplementary Methods

Materials: The solution of poly(vinyl)pyrrolidone (PVP) stabilized silver (Ag) nanocubes was purchased from nanoComposix (Cat. No. SKU:SCPH75-5M, nanoComposix Inc., San Diego, CA, USA). The average edge length of the Ag nanocubes was 75 nm. The stock nanocube concentration was ~2×10^{11} nanocubes per mL. The gold solutions were prepared from gold (III) chloride trihydrate, $HAuCl_4$ (Cat. No. 520918-5G, Sigma-Aldrich Co., St Louis, MO, USA) and gold (I) chloride, AuCl (Cat. No. 481130-1G, Sigma-Aldrich Co., St Louis, MO, USA) that were used without further purification.

Experiments: 50 µL of the Ag nanocubes solution was first transferred from the stock solution into a 1.5 mL centrifuge tube. Then, the solution was centrifuged at 10000 rpm for 5 minutes. Next, the supernatant was removed, and the remaining Ag nanocubes were re-dispersed in 50 µL deionized water to reduce the concentration of PVP in the solution.

For *in situ* experiments, we used liquid cells comprising of two chips: (1) a heater chip with a 50-nm-thick silicon nitride (SiN_x) membrane window (Hummingbird Scientific, Lacey, WA, USA) and (2) a custom fabricated bottom chip[1] with a 25-nm-thick SiN_x window (Supplementary Figure 1). The heater chips have a Mo thin film heating element embedded within the SiN_x. These chips were first plasma treated prior to experiments to make their SiN_x membrane surfaces hydrophilic. Next, approximately 500 nL of the nanocube solution was drop-casted onto the heater chip and allowed to dry. The heater and bottom chips were put together and sealed within a Hummingbird Scientific Liquid Flow holder. The entire assembly was transferred to a JEOL2010FEG transmission electron microscope (TEM) (JEOL Ltd., Tokyo, Japan). The TEM was operated at 200 kV for *in situ* imaging with a low dose rate regime where the incident electron flux ranged from 10 to 30 e^- per ($Å^2 \cdot s$). The images were acquired at a rate of 25 frames per second using the OneView CMOS camera (Gatan, Inc., Pleasanton, CA, USA). First, the heating chip had to be calibrated using software and parameters provided by Hummingbird Scientific. The temperature was calculated based on the resistivity of the heating element, where the measurement accuracy was about ±10 °C. Then, Ag nanocubes were heated to a desired experimental temperature before flowing in 1.0 mM $HAuCl_4$ solution through fluid tubing at a rate of 20 µL per min using a syringe pump. The reaction was followed in real-time. It usually takes a couple of minutes before we observe visible changes in Ag nanocubes. In a typical experiment, a few movies could be recorded in sequence by moving to fresh areas in the membrane window where nanocubes had not undergone reaction.

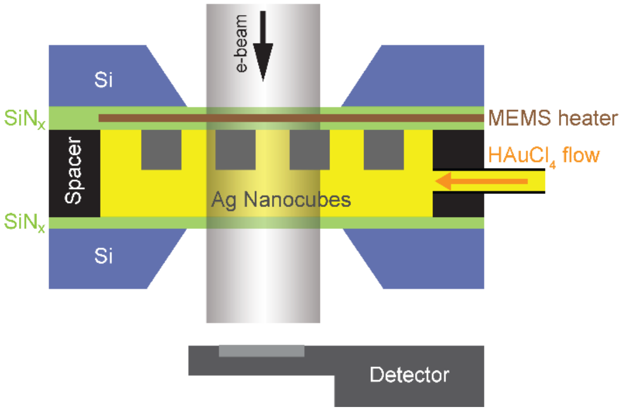

Supplementary Figure 1. Schematic of *in situ* transmission electron microscopy (TEM) setup. The liquid cell is made up of two chips that have silicon nitride (SiN_x) membrane windows. The two chips are stacked on top of each other, where the chip with the heating element is placed at the top of the stack.

Stability of Ag nanocubes inside the liquid cell during heating: Supplementary Figure 2 shows the time-lapsed TEM images of an individual Ag nanocube in water inside a heating liquid cell before introducing the $HAuCl_4$ solution. The Ag nanocube was stable, *i.e.*, retained its cuboid shape without significant degradation or changes after heating at ~90 °C for at least 3 minutes under a typical imaging condition.

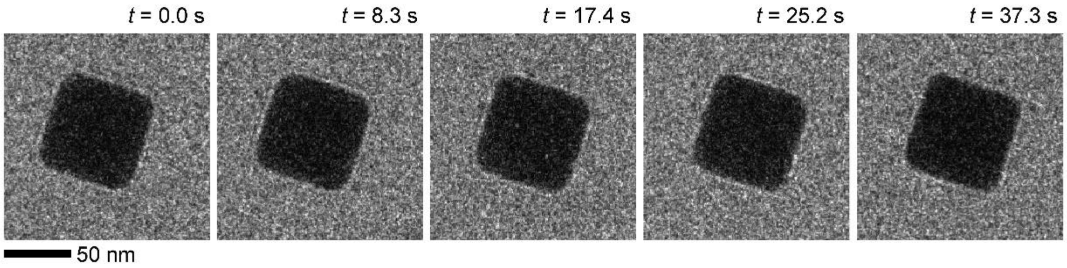

Supplementary Figure 2. *In situ* TEM time series images of an Ag nanocube in water inside a heated liquid cell held at 90 °C.

Ex situ experiments: Supplementary Figure 3 shows TEM images of Au-Ag nanostructures after titrating the aqueous nanocube solution (100 µL of the stock solution with ~2×10^{11} nanocubes per mL was added into 5 mL of PVP (Cat. No. 856568-100G, Sigma-Aldrich Co., St Louis, MO, USA)) with different amounts of aqueous $HAuCl_4$ solution at 100 °C. Consistent with previous work on this system,[2] we observed a steady increase in the size of voids within the nanostructures with increasing amount of $HAuCl_4$ added (Supplementary Figure 3).

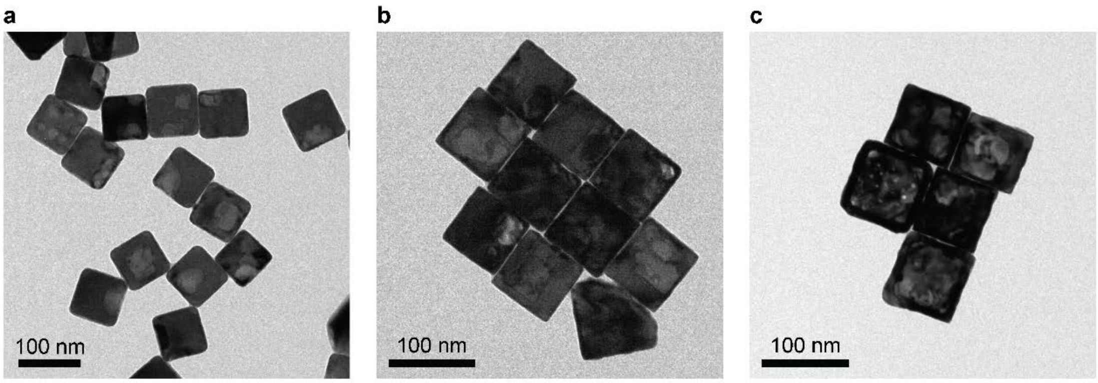

Supplementary Figure 3. TEM images of *ex situ* prepared Au-Ag nanostructures after reacting with (**a**) 0.3 mL, (**b**) 0.6 mL and (**c**) 1.5 mL of 0.1 mM $HAuCl_4$ aqueous solution at 100 °C.

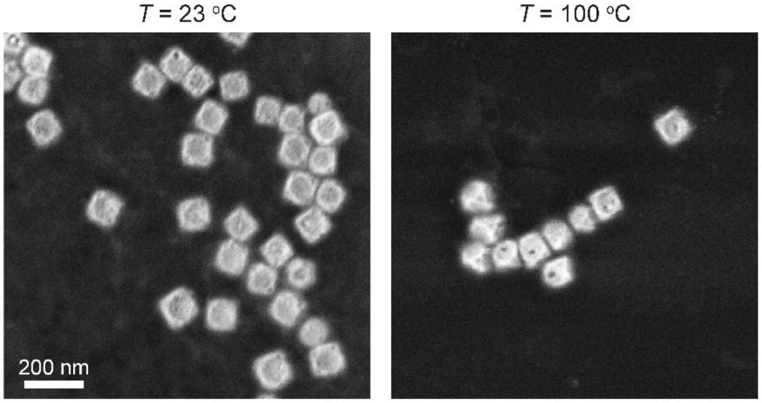

Supplementary Figure 4. SEM images of *ex situ* prepared Au-Ag nanostructures after reacting with (*left*) 1.5 mL of 0.1 mM $HAuCl_4$ aqueous solution at 23 °C and (*right*) 1.5 mL of 0.1 mM $HAuCl_4$ aqueous solution at 100 °C.

Scanning TEM (STEM) and energy dispersive x-ray spectroscopy (EDX) of particles synthesized *ex situ*: The STEM imaging and chemical analysis was carried out on an FEI Titan TEM (FEI Company, Hillsboro, OR, USA) with a Schottky electron source operated at 200 kV and an EDAX Tops System (EDAX Inc., Mahwah, NJ, USA) with a detector size of 30 mm^2. The electron probe had an approximate diameter of 0.3 nm, and the images were collected using the high angle annular dark field (HAADF) detector. A 0.5 nm electron probe and an acquisition time of 150 ms were used to collect each spectrum.

Scanning electron microscopy (SEM) of particles synthesized *ex situ*: The SEM images were acquired using an FEI Verios 460 field emission SEM operated at 2 kV and with a probe current of 50 pA. Supplementary Figure 4 shows SEM images of the nanostructures obtained at synthesis temperatures of 23 °C and 100 °C after adding 1.5 mL of 0.1 mM $HAuCl_4$ aqueous solution.

Image processing: The images presented in the manuscript figures were processed using Gatan GMS 3.0 (Gatan Inc., Pleasanton, CA, US). We performed drift correction followed by a frame averaging of 5 frames for each of the displayed images to improve the signal to noise, except for Figure 1b and Supplementary Figure 11 where 11 frames were averaged for each image. The supplementary movies were not frame averaged, and were kept at their original frame rate of 25 frames per second. They are compressed using an open-source video processing software, VirtualDub, (version 1.10.4.0) and a XVID MPEG-4 codec (Version 1.34, XVID Solutions, Germany) to reduce them to a size suitable for uploading.

To obtain the etching rates of the nanocubes shown in Figure 3b, the image sequences in the movies need to be processed and segmented. All image processing algorithms in this work were implemented in Python 2.7,[4] using the libraries, numpy,[5] scipy,[5] opencv,[6] scikit-image[7] and matplotlib.[8] In the image analysis, we performed two segmentation steps. First, we had to identify the particles in the movies. The original images in the sequences were normalized to 8-bit grayscale (0 to 255), inverted and then blurred using the Gaussian filter (scipy library[5]) employing a kernel size of $\sigma = 5$ pixels. Next, a new set of images was generated by applying a morphological black top-hat (also called as bottom-hat) transform to the blurred images. The bottom-hat transform[9] enhances dark areas of the image, hence increasing the contrast of the voids in the particles. A disk with radius $R = 99$ pixels was used as the structuring element for this operation. Then, Otsu thresholding algorithm[10] was applied to the blurred images to obtain binary images with particles being the foreground. Lastly, empty spaces in the binary objects were filled.

A second segmentation was used to extract the void/core regions of the particle. Due to the weak initial contrast of the voids, we summed five consecutive frames to increase the signal-to-noise ratio. For the sum operation, we had to perform drift correction for the consecutive five frames. We first calculated the particles' centroid positions using the binary images that were acquired earlier. Then, by comparing centroid-to-centroid distances for every two neighboring frames, we were able to classify the particles and track their trajectories. Next, we used the centroid positions to generate cutouts of the refined images that were obtained after the bottom-hat transform. Next, the five cutouts are summed, and intensity-based Li thresholding algorithms[11,12] were used to segment out the voids for the elevated temperature experiments (70 °C and 100 °C). The projected areas of the Ag core as a function of time, as provided in Figure 3b, were obtained by subtracting the size of the voids from the original size of the nanocube.

For the data set of experiments at 23 °C (Figure 1a), it was more straightforward to define the Ag core. In this case, the cut-outs and their sums were generated from the blurred images. Next, the Otsu thresholding algorithm[10] was used to extract the particle from the background. Then, this binary image

was used as the mask to extract the intensities inside the particle's boundary. A second Otsu thresholding was executed on this set of intensities to segment the core of the particle, and the projected area was obtained directly. However, the dissolution rates were difficult to quantify in general because of a significant variation across different nanocubes under the same conditions. This issue arises because we are using the projected TEM images to extrapolate volumes. In TEM, the image is a 2-dimensional projection of a 3-dimensional object. Therefore, depending on the direction in which the void propagates, the apparent dissolution can be different (for example, see Supplementary Figure 8).

Supplementary Note 1: Chemical Characterization of Nanostructures Obtained by Galvanic Replacement at Different Temperatures

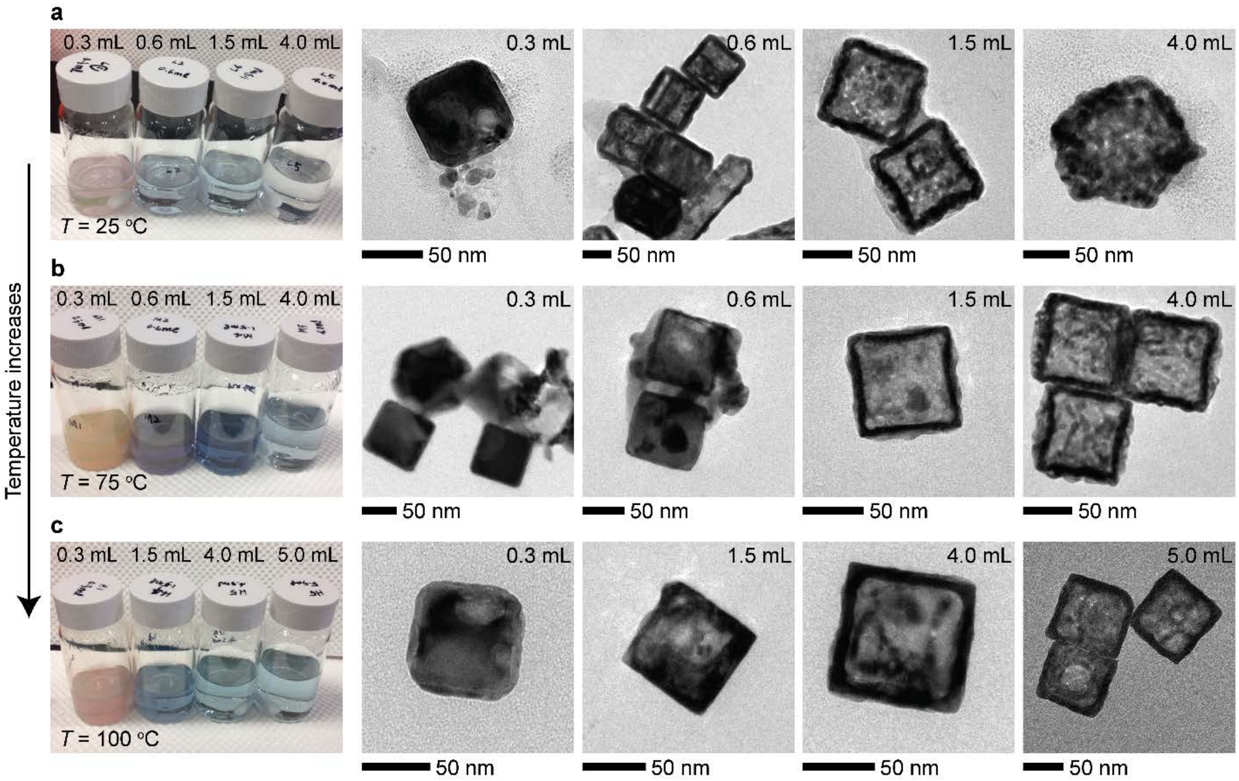

Supplementary Figure 5. The first column of photograph panels shows the changes in solution color observed during the galvanic reaction at different temperatures: (**a**) ~25 °C, (**b**) ~75 °C and (**c**) ~100 °C and after adding 0.3 mL to 5.0 mL of 0.1 mM $HAuCl_4$ aqueous solution to the nanocube solution (100 µL of the stock solution with ~2×10^{11} nanocubes per mL into a reaction flask that holds 5 mL of PVP). The reacting solution was left to stabilize for 10 minutes after $HAuCl_4$ addition and before the samples were extracted. The 2nd to 5th columns of panels show TEM images of the *ex situ* prepared Au-Ag nanostructures after reacting with different amount of 0.1 mM $HAuCl_4$ aqueous solution at corresponding temperatures.

For comparison with *in situ* observations, we reacted the Ag nanocubes solution *ex situ* with different amounts of $HAuCl_4$ at different temperatures (25 °C, 75 °C and 100 °C, see Supplementary

Figure 5). Color changes in the solutions were consistent with the earlier reports.[3] After cooling to room temperature, these nanostructures were washed by adding saturated NaCl solution to the reaction mixture followed by two rounds of centrifugation and re-dispersion in deionized water. The NaCl wash removed AgCl that was present in the reaction mixture.[3] At lower temperatures (25 °C and 75 °C), we often found rough Au-Ag nanoboxes, which compares well to Figure 1a at $t - t_0 = 4.0 - 12.0$ s. At 100 °C; Au-Ag nanoboxes with relatively smooth and homogeneous walls were formed.

We also performed EDX mapping of these *ex situ* prepared Au-Ag nanostructures. EDX allows us to map out the elemental composition of these nanostructures through the identification of characteristic X-rays emission from the sample. Supplementary Figure 6 shows the high-angle annular dark-field STEM images of nanostructures synthesized at different temperatures and their corresponding STEM-EDX chemical maps. From these maps, it is clear that the Au mainly deposits on the surfaces of the structures and forms an Au-Ag alloy.

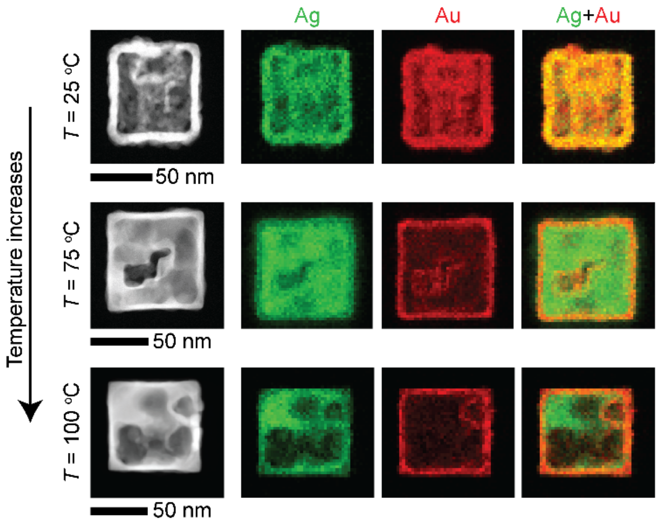

Supplementary Figure 6. STEM images and the corresponding STEM-EDX chemical maps of washed nanostructures synthesized in the test tube at different temperatures after reacting them with 1.5 mL of 0.1 mM $HAuCl_4$ aqueous solution.

Supplementary Figure 7a-c shows the HAADF STEM images and EDX maps of nanostructures synthesized *ex situ* at different temperatures from supported Ag nanocubes. Here, we dispersed the nanocubes on TEM grids (TEMWindows.com, West Henrietta, NY, USA) with SiN_x windows. Then, the samples were heated on a hotplate to their respective temperatures before 1 mM aqueous $HAuCl_4$ was dropped onto them. All samples showed clear signatures of Cl on the nanostructure surface, where the Cl content dropped from ~40% (at.) at 23 °C to ~8% (at.) at 90 °C. The appearance of solid AgCl is consistent with the reaction chemistry and with the solubility of AgCl in water decreasing at lower temperatures.[2]

Supplementary Figure 7d shows another experiment where we analyzed the nanostructures in the liquid cell after a brief reaction with $HAuCl_4$ at 90 °C. We unloaded the liquid cell once there were signs of a reaction. Then, we took apart the liquid cell and rinsed the heating chip in water. The results confirmed that the thin liquid conditions in these cells encouraged the precipitation of solid AgCl around the nanostructures (Supplementary Note 3).

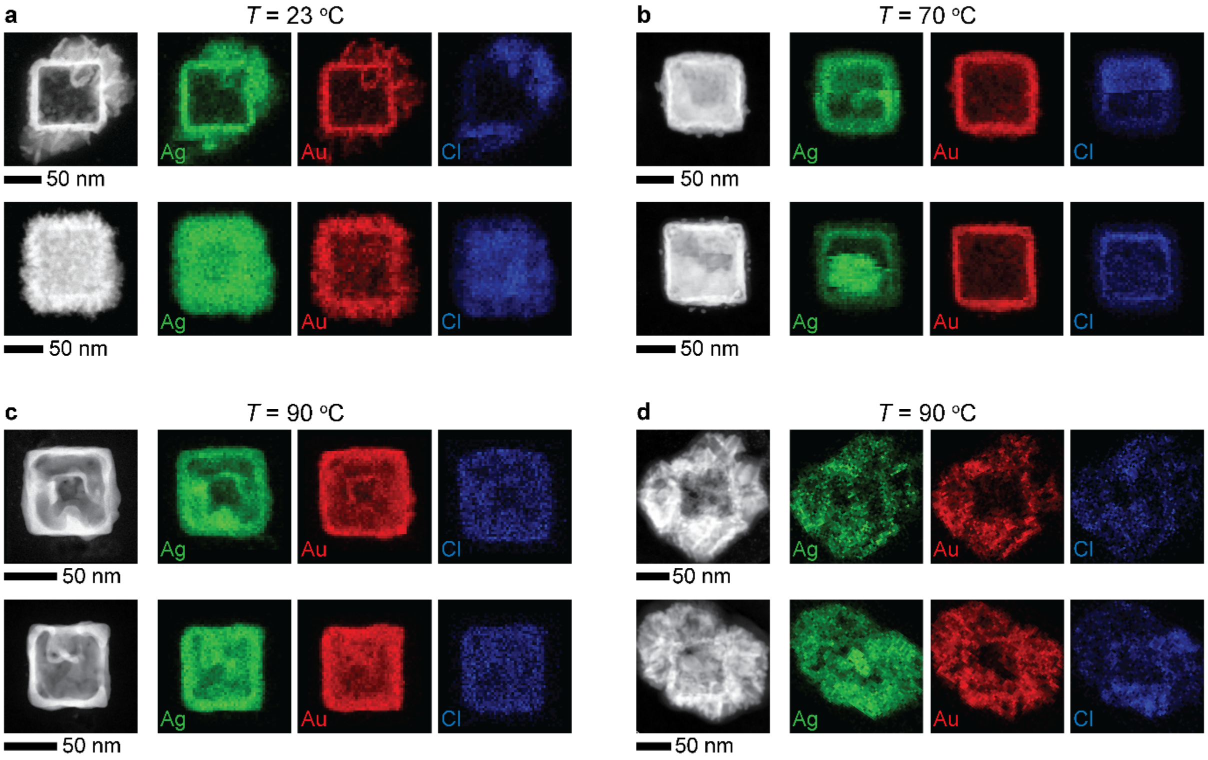

Supplementary Figure 7. STEM images and the corresponding STEM-EDX chemical maps of nanostructures after aqueous $HAuCl_4$ was dropped onto Ag nanocubes that were drop-cast on SiN_x membrane window TEM grids at (**a**) 23 °C, (**b**) 70 °C and (**c**) 90 °C. There are strong signatures of Cl on the nanostructure surfaces. In (**d**), we characterized a sample that was reacted within the liquid cell at ~90 °C, but characterized *ex situ*. The Ag nanocubes were only exposed to the electron beam for a brief duration (to confirm the reaction). In this case, we see a significant amount of AgCl on the surface of the nanostructures.

Supplementary Note 2: Chemical Effects due to the Radiolysis by the Electron Beam

The primary by-products generated by the radiolysis of water are solvated electrons (e^-_{aq}), hydroxyl free radicals (OH•), hydrogen free radicals (H•), and molecular hydrogen (H_2), where the first two products are the dominant reactive species.[13] In this case, the solvated electrons are strongly reducing species (which often exploited to initiate the nucleation of metal nanoparticles in liquid cell TEM[14–16]), whereas the hydroxyl free radicals are highly oxidizing (reduction potential of +2.7 V versus the standard hydrogen electrode in acidic solutions). The overall effect on the imaged specimen depends on the experimental parameters. We highlight here the electron flux used in our current work is significantly

lower than what we previously used to induce Au nucleation (800-8000 e^- per (Å^2·s)).[16] The electron flux of 30 e^- per (Å^2·s) is considered low for *in situ* TEM experiments. It had already been reported that this flux did not induce the nucleation of Au nanocrystals.[17,18] Comparatively, the threshold electron flux for nucleation and growth of Ag nanocrystals had been reported to be 0.5 e^- per (Å^2·s).[19] Here, we decreased the flux by 3 times to about 10 e^- per (Å^2·s) to check if there were obvious signs of beam induced effects (Supplementary Figure 8). There were no significant differences in the observed morphologies between images below and those recorded at higher electron fluxes (Figure 1b).

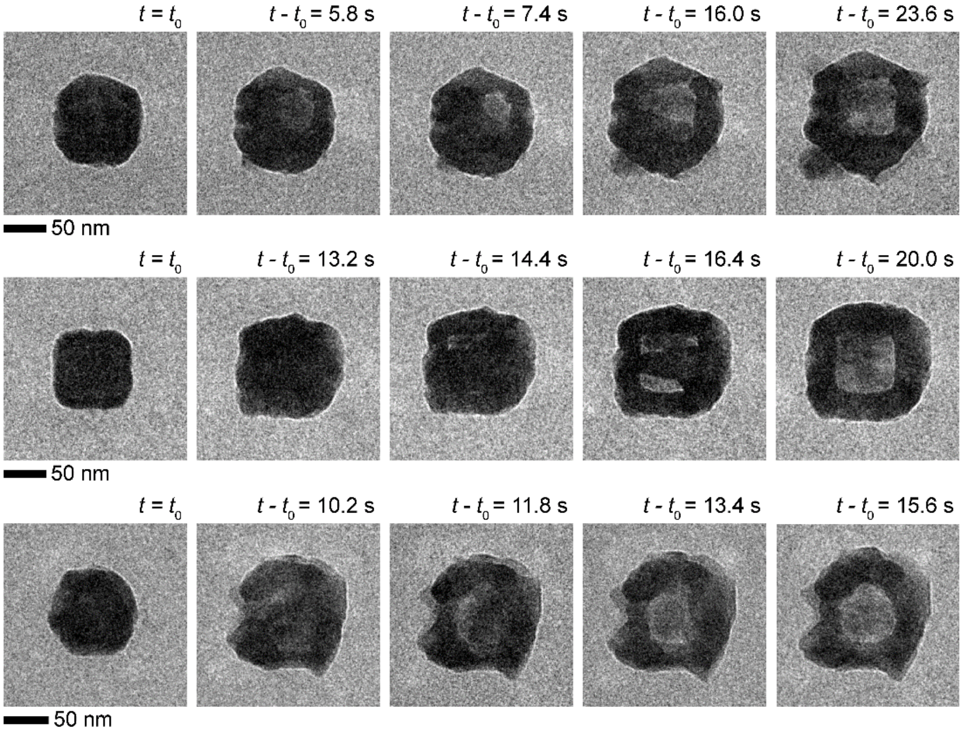

Supplementary Figure 8. *In situ* TEM time series images showing sequences of galvanic replacement reaction for three different nanocubes recorded at 90 ºC with electron fluxes of 10 e^- per (Å^2·s).

It had also been suggested that OH• may influence galvanic replacement reactions by greatly accelerating the oxidation of Ag, leading to a significant difference in time scales between *in situ* and *ex situ* results pertaining to reactions between Ag and Pd[20] (reactions taking place within a minute in a liquid cell versus hours in a benchtop synthesis). While it is possible that the electron beam also accelerated the oxidation of Ag in our experiments involving the replacement reaction between Ag and Au, we do not find a large discrepancy in reaction times between the *ex situ* and *in situ* experiments. Although we had to wait 10 minutes for the reaction mixture to stabilize after $HAuCl_4$ addition in *ex situ* synthesis, the nanostructures extracted from those solutions (Supplementary Figure 5 and 6) would have completed their

transformation well within that time frame. The time scale of reactions we observed *in situ* was also consistent with the transitions times of a few seconds observed by Smith *et al.*[21] using *in situ* plasmon spectroscopy. Lastly, Lewis *et al.*[22] had reported that the electron beam could induce transformations of AuAg alloy nanoparticles in conventional TEM that appears similar to the nanoscale Kirkendall effect. However, the electron flux required to trigger the transformation was 2000 e^- per ($Å^2$·s), which is ~100× more than what was used in our experiments.

Supplementary Note 3: Solubility of AgCl at 100 °C under Thin Liquid Conditions

Here, we compare the amount of AgCl that is produced from the galvanic replacement of the Ag by Au against the expected solubility of AgCl at 100 °C in water. Assuming an Ag nanocube with an edge length of 75 nm is completely dissolved in a solution containing Cl^- ions, the number of Ag ions that will be produced is 2.5×10^7 (calculated using the bulk Ag density of 10.49 g per cm^3 and molar mass of 107.9 g per mol for Ag). The solubility product, K_{sp} for AgCl is given by

$$K_{sp} = [Ag^+][Cl^-] \quad (1)$$

where Sun and Xia[2] had reported a K_{sp} of 1.2×10^{-6} mol^2 per L^2 for AgCl at 100 °C. Assuming all the Ag^+ ions react with Cl^- ions to form AgCl, there will be $\sim4\times10^{-17}$ moles of AgCl present in the solution. It means that ~0.04 pL of water is needed to dissolve all that AgCl, which translates to $\sim4\times10^{10}$ nm^3 or a liquid volume that is about 3 µm × 3 µm × 3 µm. This condition is not fulfilled in our liquid cells experiment where the liquid layers are usually only a few hundred nanometers thick.[23] In Supplementary Figure 9, we show that an experiment where some of the dissolved AgCl precipitated out at later times around the nanocube.

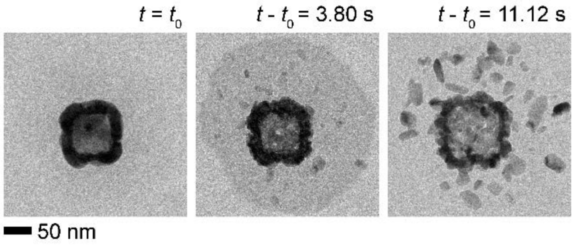

Supplementary Figure 9. *In situ* TEM time series images showing the precipitation of AgCl around the Ag nanocube on the SiN_x membrane recorded at ~85 ºC.

We also tried to control the formation of AgCl by dispersing the nanocubes in ethylene glycol. In this case, the nanocube solution ($\sim2\times10^{11}$ nanocubes per mL) was re-dispersed in a mixture of 1% (v/v) ethylene glycol in water, rather than water as described in the methods earlier (Supplementary Methods).

Supplementary Figure 10 depicts a time series of the reaction observed at 90 °C. In this case, it appears that the presence of ethylene glycol suppresses the formation of solid AgCl[24] and we see an uneven layer of lighter contrast surrounding the nanocube (Supplementary Figure 10a: $t - t_0$ = 5.0 to 40.0 s). Furthermore, the nanocube dissolution appears to occur in two stages. The first stage involves the formation of an initial cavity (Supplementary Figure 10a: $t - t_0$ = 5.0 s, Supplementary Figure 10b: $t - t_0$ = 12.0 s), which may be associated with the commonly proposed pinhole dissolution mechanism.[2] However, the cavities do not continue to expand (Supplementary Figure 10a: $t - t_0$ = 10.0 s). After several seconds, we see again hollowing of the entire nanostructure via isotropic outward diffusion of Ag. This transformation leads to the formation of a nanocage (Supplementary Figure 10a: $t - t_0$ = 40.0 s, Supplementary Figure 10b: $t - t_0$ = 140.0 s).

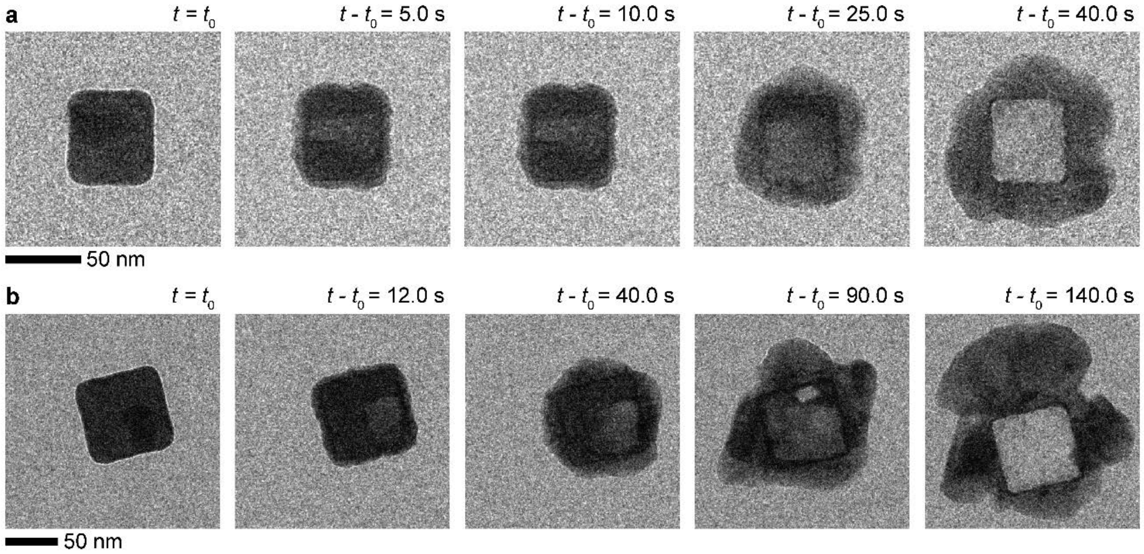

Supplementary Figure 10. *In situ* TEM time series images showing the sequences of galvanic replacement reaction for two different nanocubes dispersed in a mixture of ethylene glycol and water, recorded at 90 °C.

Supplementary Note 4: *In Situ* Observations of Nanocage Formation through Etching with $Fe(NO_3)_3$

Hollow nanocages can also be formed by using an Ag etchant such as iron nitrate, $Fe(NO_3)_3$, to remove Ag from partially reacted nanostructures that had undergone GR.[25] In contrast to the reaction with $HAuCl_4$, this procedure only induces Ag dealloying and de-couples it from Au deposition. Supplementary Figure 11a reveals how Ag was removed from such nanostructures when 0.1 mM $Fe(NO_3)_3$ solution (made using iron (III) nitrate nonahydrate, Cat. No. 216828-100G, Sigma-Aldrich Co., St Louis, MO, USA) was introduced *in situ* at 23 °C. Here, the *ex situ* synthesized nanostructures after 0.6 mL of 0.1 mM $HAuCl_4$

had been added (at 100 °C) were drop-casted on the liquid cell chips. Then, $Fe(NO_3)_3$ solution was flowed into the liquid cell through the fluid tubing. In this case, it appears that the dissolution progresses *via* void propagation where the initial void formation is clearly seen at $t - t_0$ = 37.0 s and the nanocage formation at $t - t_0$ = 125.0 s. The final shape of this nanocage is consistent with the cages formed *ex situ* (Supplementary Figure 11b).

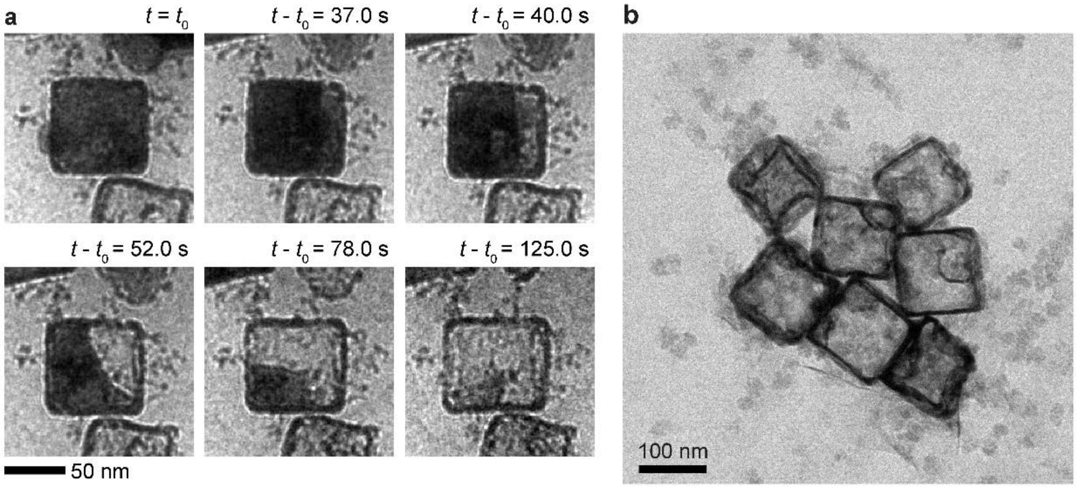

Supplementary Figure 11. (**a**) *In situ* TEM time series images showing complete hollowing of Ag nanostructures at 23 °C using 0.1 mM $Fe(NO_3)_3$ as an etchant. (**b**) TEM images of *ex situ* prepared Au nanoframes by using solution in (c) to react with 0.25 mL of 1.0 mM of $Fe(NO_3)_3$ aqueous solution.

Additional Supplementary Figures

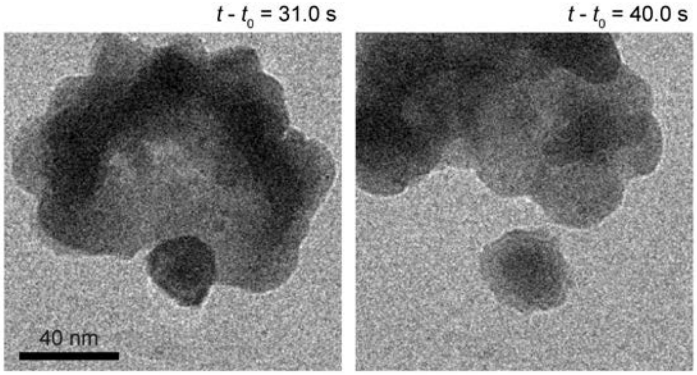

Supplementary Figure 12 Inserts from Supplementary Movie 3 depicting galvanic replacement of the residual Ag core once the outer shell detaches from the membrane surface.

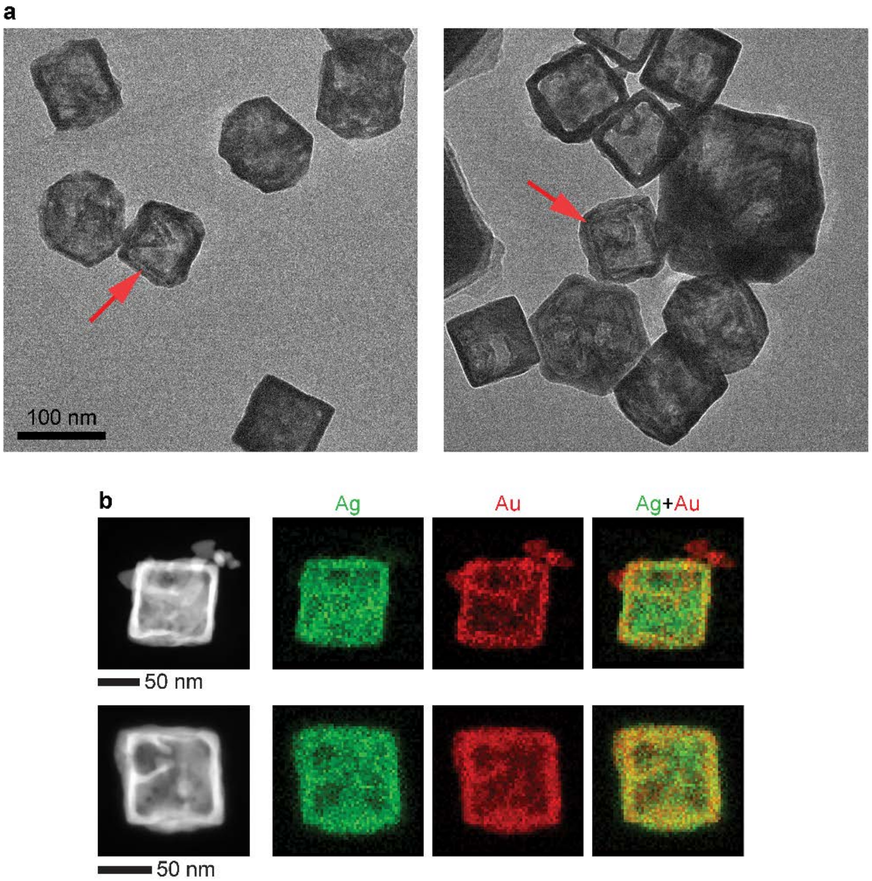

Supplementary Figure 13. (**a**) Double wall formation observed in nanostructures synthesized *ex situ* using aqueous AuCl (5.5 mL of 0.1 mM AuCl added to 100 µL of nanocube solution) at 100 °C. (**b**) STEM images and EDX maps of two nanostructures synthesized with AuCl.

Supplementary References

1. Liu, Q. *et al.* Nanodroplet depinning from nanoparticles. *ACS Nano* **9,** 9020–9026 (2015).
2. Sun, Y. & Xia, Y. Mechanistic study on the replacement reaction between silver nanostructures and Chloroauric Acid in Aqueous Medium. *J. Am. Chem. Soc.* **126,** 3892–3901 (2004).
3. Skrabalak, S. E., Au, L., Li, X. & Xia, Y. Facile synthesis of Ag nanocubes and Au nanocages. *Nat. Protoc.* **2,** 2182–2190 (2007).
4. Oliphant, T. E. Python for scientific computing. *Comput. Sci. Eng.* **9,** 10–20 (2007).
5. Van Der Walt, S., Colbert, S. C. & Varoquaux, G. The NumPy array: A structure for efficient numerical computation. *Comput. Sci. Eng.* **13,** 22–30 (2011).
6. Bradski, G. The OpenCV library. *Dr Dobbs J. Softw. Tools* **25,** 120–125 (2000).
7. van der Walt, S. *et al.* scikit-image: image processing in Python. *PeerJ* **2,** e453 (2014).
8. Hunter, J. D. Matplotlib: A 2D graphics environment. *Comput. Sci. Eng.* **9,** 99–104 (2007).
9. Serra, J. P. *Image analysis and mathematical morphology*. (Academic Press, 1983).
10. Otsu, N. A threshold selection method from gray-level histograms. *IEEE Trans. Syst. Man. Cybern.* **9,** 62–66 (1979).
11. Li, C. H. & Lee, C. K. Minimum cross entropy thresholding. *Pattern Recognit.* **26,** 617–625 (1993).
12. Li, C. H. & Tam, P. K. S. An iterative algorithm for minimum cross entropy thresholding. *Pattern Recognit. Lett.* **19,** 771–776 (1998).
13. Schneider, N. M. *et al.* Electron–water interactions and implications for liquid cell electron microscopy. *J. Phys. Chem. C* **118,** 22373–22382 (2014).
14. Zheng, H. *et al.* Observation of single colloidal platinum nanocrystal growth trajectories. *Science* **324,** 1309–12 (2009).
15. Woehl, T. J., Evans, J. E., Arslan, I., Ristenpart, W. D. & Browning, N. D. Direct in situ determination of the mechanisms controlling nanoparticle nucleation and growth. *ACS Nano* **6,** 8599–610 (2012).
16. Loh, D. *et al.* Multi-step nucleation of nanocrystals in aqueous solution. *Nat. Chem.* **9,** 77–82 (2016).
17. Wu, J. *et al.* Growth of Au on Pt icosahedral nanoparticles revealed by low-dose in situ TEM. *Nano Lett.* **15,** 2711–2715 (2015).
18. Park, J. H. *et al.* Control of electron beam-induced Au nanocrystal growth kinetics through solution chemistry. *Nano Lett.* **15,** 5314–5320 (2015).
19. Noh, K. W., Liu, Y., Sun, L. & Dillon, S. J. Challenges associated with in-situ TEM in environmental systems: the case of silver in aqueous solutions. *Ultramicroscopy* **116,** 34–8 (2012).
20. Sutter, E. *et al.* In situ liquid-cell electron microscopy of silver-palladium galvanic replacement reactions on silver nanoparticles. *Nat. Commun.* **5,** 4946 (2014).
21. Smith, J. G., Yang, Q. & Jain, P. K. Identification of a critical intermediate in galvanic exchange reactions by single-nanoparticle-resolved kinetics. *Angew. Chemie - Int. Ed.* **53,** 2867–2872 (2014).
22. Lewis, E. A. *et al.* Real-time imaging and elemental mapping of AgAu nanoparticle transformations. *Nanoscale* **6,** 13598–13605 (2014).
23. Chee, S. W., Baraissov, Z., Loh, N. D., Matsudaira, P. T. & Mirsaidov, U. Desorption-mediated motion of nanoparticles at the liquid–solid interface. *J. Phys. Chem. C* **120,** 20462–20470 (2016).
24. Trimble, H. M. Solubilities of salts in ethylene glycol and in its mixtures with water. *Ind. Eng.*

Chem. **23,** 165–167 (1931).
25. Lu, X. *et al.* Fabrication of cubic nanocages and nanoframes by dealloying Au/Ag alloy nanoboxes with an aqueous etchant based on $Fe(NO_3)_3$ or NH_4OH. *Nano Lett.* **7,** 1764–1769 (2007).
