# Supplementary material for: Direct observation of the nanoscale Kirkendall effect during galvanic replacement reactions
Source: Nat Commun. 2017 Oct 31;8:1224. doi: 10.1038/s41467-017-01175-2 (PMC5663914; doi:10.1038/s41467-017-01175-2)
Supplement: Supplementary file 2 — Description of Additional Supplementary Information [file 41467_2017_1175_MOESM2_ESM.pdf]

Description of Additional Supplementary Files

File Name: Supplementary Movie 1
Description: Galvanic replacement of Ag nanocubes by Au using 1 mM $HAuCl_4$ at 23 °C recorded in real time and at 25 frames per second. The movie has been compressed from the raw images, without further processing.

File Name: Supplementary Movie 2
Description: Galvanic replacement of Ag nanocubes by Au using 1 mM $HAuCl_4$ at 90 °C recorded in real time and at 25 frames per second. The movie has been compressed from the raw images, without further processing.

File Name: Supplementary Movie 3
Description: Galvanic replacement of Ag nanocubes by Au using 1 mM $HAuCl_4$ at 70 °C recorded in real time and at 25 frames per second. The movie has been compressed from the raw images, without further processing.

File Name: Supplementary Movie 4
Description: Galvanic replacement of Ag nanocubes by Au using 1 mM AuCl at 90 °C recorded in real time and at 25 frames per second. The movie has been compressed from the raw images, without further processing.
